# Ethnobotanic, Ethnopharmacologic Aspects and New Phytochemical Insights into Moroccan Argan Fruits

**DOI:** 10.3390/ijms18112277

**Published:** 2017-10-30

**Authors:** Farid Khallouki, Mohamed Eddouks, Akdad Mourad, Andrea Breuer, Robert Wyn Owen

**Affiliations:** 1 Divisionof Preventive Oncology, National Center for Tumor Diseases, Im Neuenheimer Feld 460, German Cancer Research Center (DKFZ), Im Neuenheimer Feld 581, D-69120 Heidelberg, Germany; andrea.breuer@nct-heidelberg.de; 2 Team of Physiology, Nutrition and Endocrinology, Biology Department, FSTE, BP 509 Boutalamine, Errachidia, Morocco; mohamed.eddouks@laposte.net (M.E.); akdad.mourad@gmail.com (A.M.)

**Keywords:** *Argania spinosa*, argan fruits, argan oil, botany, ethnobotany, secondary metabolites, health and environmental benefits

## Abstract

This review summarizes available data on argan fruit botany, geographical distribution, traditional uses, environmental interest, socioeconomic role, phytochemistry, as well as health beneficial effects and examination of future prospects. In particular, ethnomedical uses of argan fruits are carried out throughout Morocco where it has been used against various diseases. Different classes of bioactive compounds have been characterized including essential oils, fatty acids, triacylglycerols, flavonoids and their newly reported acylglycosyl derivatives, monophenols, phenolic acids, cinnamic acids, saponins, triterpenes, phytosterols, ubiquinone, melatonin, new aminophenols along with vitamin E among other secondary metabolites. The latter have already shown a wide spectrum of in vitro, and ex vivo biologicalactivities including antioxidant, anti-inflammatory, anti-diabetic, antihypertensive, anti-hypercholesterolemia, analgesic, antimicrobial, molluscicidal anti-nociceptive and anticancer potential. Argan flesh (pulp) contains a broad spectrum of polyphenolic compounds which may have utility for incorporation into nutraceuticals and cosmeceuticals relevant to the food, cosmetic and health industries. Further research is recommended, especially on the health beneficial effects of the aminophenols.

## 1. Introduction

The argan tree *Argania spinosa* (L.) Skeels, an endemic species of Morocco with tropical affinities, is typically a multi-purpose tree, and plays a very important socio-economic role in this country, while maintaining an ecological balance. This species is the only representative of the tropical family Sapotaceae in Morocco [1]. The tree is the second largest forest species, after oak and before cedar, andcan live up to 200 years [2]. The tree was recognized as a biosphere reserve since 1998 and was declared as a “protected species” by United Nations Educational, Scientific and Cultural Organization (UNESCO) [3].

The argan tree has very specific chemical compositions which fortify their potential in particular for use in food, cosmetic, and medical preparations. The argan tree supports the livelihood of rural populations as a source of income and therefore they depend on the aganeraie. The various botanical parts of the tree also make a large contribution to biodiversity.

All botanical parts of the tree are exploited: the wood, leaves and fruits, the kernelsof which yield argan oil, one of the rarest and most expensive oils in the world.

Argan oil has seen its popularity increase over the centuries and its therapeutic values have been reported since 1219 by the eminent Arab doctor, Ibn Baytar who was the first to write about the argan tree. Very early scientific data were conducted and some examples are given in the literature [4,5]. However, the first interesting chemical reports on argan tree products were published by Farines et al. (1981, 1984) [6,7]. Subsequently, further elegant reports [8,9,10,11,12,13,14,15,16,17,18] have been published greatly furthering our knowledge on the phytochemical composition of argan fruits.

In this review, we focus on detailed aspects of ethnobotanical and ethnomedical data, along with a comprehensive update on the phytochemical composition of the fruits. We also include new insights about natural products that have only recently been reported in the fruit of the argan tree.

### 1.1. Botanical Description

The argan tree (*Argania spinosa* skeels L., syn. *Argania syderoxylon* L., *Sideroxylon spinosum* L. and *Elaerandronargan* Retz) belongs to the Sapotaceae family, and is a unique species of this family growing in a subtropical zone.

According to Quezel and Santa (1962) [19], the argan tree belongs to: Division, Magnoliophyta; Class, Magnoliopsida (dicotyledon); Order, Ebenales; Family, Sapotaceae; Genus, *Argania*; Species, *Argania spinosa* Skeels (L.).

The tree is in the form of bushes; its silhouette is of a broad crown with a specific tortuous trunk; the branches are thorny, hence its acquired name “spinosa”; and its growth (up to 8 m) is slow. Argan tree wood is hard and heavy; andthe leaves attenuated in a short petiole and are alternate, lanceolate, usually 2 to 3 cm long, dark green on the upper side, and lighter below [20].

The flowers appear at the end of spring and lead to fruits that reach maturity around September. The white to greenish-yellow flowers are hermaphrodite gamopetals with a very short tube and are combined into glomeruli. These appear in May–June. The fruit is a false drupe, oval sessile bay, fusiform, rounded or in a spindle and is about 4 cm long with a very hard nut containing two or three kernels representing about a quarter of the weight of the fresh fruit. A tree produces about 8 kg per year. The fruit color is greenish before maturation, and then it evolves towards yellow or light-yellow brown. A dark brown or black color develops after abscission of the mature fruit. The fruit is made up of a fleshy pericarp (pulp) which ripens in the spring and falls to the ground in early summer [21,22,23,24].

### 1.2. Geographical Distribution

The area of the argan tree probably covered 1,400,000 ha at the beginning of the quaternary era. However, it was reduced to 700,000 ha by 1965 [22,23], and now three-quarters of the argan forest is in the regions of Essaouira and Agadir. Nowadays, the argan tree is widespread, in the arid and semi-arid regions of southwestern Morocco and covers about 870,000 ha, representing now only about 17% of the original Moroccan forest area, which amounts to twenty million trees, growing essentially in the provinces of: Essaouira, 130,000 ha; Agadir, 37,000 ha; Chtouka-Aït Baha, 90,000 ha; Tiznit, 140,000 ha;Taroudant, 360,000 ha; and Inzeguane-Aït Melloul, 13,000 ha [25].

More specifically, the tree extends from the mouth of the valley Tensift in the north, to the valley Drâa in the south and is also encountered in the Souss plain, on the southern slope of the western high Atlas Mountains and on the northern and southern slopes of the western Anti-Atlas Mountains at altitudes between 1300 and 1500 m. Inland, the distribution of the argan tree extends to the region of Taroudant. Beyond this geographical location, two other small areas of argan tree are recorded in the upper Grou valley in the southeast of Rabat and in the northwestern foothills of the Beni Snassen, near Oujda [26,27,28]. The argan tree has been introduced as a cultivated species in the deserts of Tunisia, Israel, and South Africa, among other parts of the world [29].

### 1.3. Environmental Interest of the Argan Tree

The argan tree is a polymorphous, thermophilic and xerophytic species, growing in arid and semi-arid bioclimates. Argan tree roots develop deeply, helping prevent wind erosion and desertification of the soil, in addition, owing to their deep-growing roots, trees shade different types of crops, and help maintain soil fertility through evaporation. The flora that cohabits with it is also important. Adult trees can grow in temperatures up to 50 °C, can evolve in most soil types but suffer if soil is not well drained. However, the tree is not suitable for very high altitudes or high salinity [30]. One third of Morocco’s vegetation, including several endemic species, is found in the Arganeraie area. Researchers have already recorded 100 plant species growing near the argan tree [31]. Consequently, the disappearance of the argan tree inevitably leads to the disappearance of many other species as well, leading to a reduction of the genetic inheritance of the tree itself as well as for other animal and plant species.

### 1.4. Economic Interest

The Arganeraie constitutes an important source of income for the Moroccan Berber populations. The press cake is used for fattening cattle, while fruit pulp and leaves also constitute a fodder for animals. The wood of the argan tree is extensively used as an energy bioresource, in the form of coal. The most economically viable part of the tree is its fruit, which provides food and cosmetic oils. The global demand for this oil is now increasing in the North American, European Union, Asia Pacific (China and Japan), Middle East andSouth African markets. The number of personal-care products on the US market including argan oil as an ingredient increased from just two in 2007, to over one hundred by 2011 [32].

The argan tree has created many jobs through the creation of women’s cooperatives. The global argan oil market was 4835.5 tons in 2014 and is expected to reach 19,622.5 tons by 2022 [33].

### 1.5. Ethnobotany

The first description of the argan tree was made in the 13th century by the Egyptian Doctor Ibn Al-Baytar. It is nicknamed “tree of life”but has several other vernacular names: iron tree, al’argane or more rarely olive tree of Morocco. In 1515, El Hassan ben Mohamed el Wazzani Ezagyati (known as Jean Leon L’africain) described the tree as a spiny species with an oleagineous oil. In 1737, Linne classified the argan tree in the genus *Rhammus* under the name *Sideoxylon spinosun* L. In 1819, Roem created the genus *Argania* with a monotype *Argania spinosa*, belonging to the Sapotaceae family [34].

Following a fuel crisis in 1917, destruction of thousands of hectares of argan tree was reported [35]. Consequently, the argan tree is now owned by the Moroccan state, where the inhabitants of the region retain the right to crop and benefit from the forest [36].

The argan tree, is a surviving relic species with very hard and heavy red wood used mostly in the manufacture of tools, production of decoratively inlaid boxes, in carpentry and construction materials as well as in the production of coal.Its short trunk allows goats to climb along its branches to feed on its botanical parts (foliage and fruits). The fruit is rich in oil, which is used as an alimentary and for cosmetic or pharmaceutical purposes. Fruits, leaves and the residue of oil extraction (zikmum) are a source of food for fattening animals. Fruit production varies with tree age, density, rainfall, maturity, growing conditions, storage conditions, fertilizer, soil type, season, geographic origin and amount of sunlight exposure. Overall, production of 100 kg of ripe fruit yields 60 kg of dried matter giving approximately 30 kg of dry pulp and 30 kg of hard core.

Etymologically, the name argan comes from the Berber word “arjan” which in the current language has evolved into “Argan” or “arqân”. This term probably derives from “rajnah” which means “stay locked in one fixed place”. Indeed, argan is a native and curiously an endemic tree of Morocco, where it remains confined in a localized area otherwise. However, it has been introduced into the deserts of Israel, Algeria among other world regions.

The word “arqân” shows a transformation language of “arjan”, or by the use of its oil as a cosmetic product, there seems to be a reconciliation of this product to the term “al-arqân” which means in Berber, leaves of l’henna (*Lawsonia inernis*) which is also used in cosmetics to make hair more beautiful in Morocco, and also as a healing of skin wounds, skin dryness as well as other skin physiological aging symptoms, here there is a common traditional use of l’henna and argan oil.

The argan tree is a typical example of ethnobotany based on three main components: a tree, man and goat (=“arad”, for the male and “o’tatat”for the female). For the local population, the arganeraie domain is divided into temporary enclosures (=“akal”), in which local populations work during the summer harvest season only (personal Information).

The oil is beneficial against deafness and chronic earache. To tighten the abdomen, it is given in doses of one drachma; it is also traditionally used in anointing the hair, in the treatment of dry and wrinkled skin, against acne, chapped skin and burns, juvenile acne, eczema, varicella or dermatoses and to prevent stretch marks in pregnant women. In addition, it is used in the preparation of “almandaq”, a food tonic made with semolina, barley and argan oil for children especially.

In addition, the oil is used in aphrodisiac preparations such as in the preparation of “AMLO” or “âmlû”which is a mixture of toasted and ground almonds and argan oil, or for the preparation of “Bsis”, a mixture of barley flour, honey and argan oil which is considered among the inhabitants as a means of fortifying food.

Moreover, it is used extensively in culinary preparations especially in the South of Essaouira which prepares the so-called “taguramt”, consisting of argan oil, bread, smoked meat (=“HLI”), honey and butter.

Other ethnopharmacological data include treatment against rheumatism, blood circulation problems as well as the treatment of Moroccan patients with atherosclerotic disease and high blood cholesterol.

## 2. Phytochemical Studies of the Argan Fruits

### 2.1. Argan Oil Chemistry

#### 2.1.1. Extraction

Historically, extraction of argan oil was done manually, and was conducted by women. Nuts were broken with rocks and the kernels, air-dried in clay containers and roasted for food argan oil production or unroasted in the case of cosmetic oil preparation. Nowadays, this extraction is mechanized in Women’s Cooperatives developed by Charroufs Group in Morocco. The pulping of the fruits is also carried out using scraper pulping machines; virgin argan oil is produced by cold pressing of the previously roasted (food argan oil) and unroasted (cosmetic oil), respectively. These processes considerably reduce production time, to achieve a yield greater than 45% (*v*/*w*) and to guarantee a reproducibility of extraction of argan oil of very good quality [37].

According to Matthäus and Spenser (2008) [38], who have clarified the difference between cold-pressed oils and virgin oils, argan oil can be cold-pressed (without application of heat in the mechanical process) and has in addition a weak acidity index due to its slightly roasted kernel which therefore can be deemed as virgin oil.

#### 2.1.2. Argan Oil Fatty Acids

The profiles of the long-chain fatty acids in argan oil are predominated by unsaturated oleic acid (C18–1, n-9) closely followed by linoleic acid (C18–2, n-6) [16] among other types of fatty acids confirmed in trace levels in previous studies (11). On average, of the saturated long-chain fatty acids, palmitic acid (C16–0) predominates with minor amounts of stearic acid (C18–0), representing 16% and 5.5% of the total oil fatty acids, respectively [16].

#### 2.1.3. Argan Oil Triglycerides

Triacylglycerol molecular species of argan oil include three oleic (O, O, O) (12%), two linoleic and one oleic (L, L, O) (13.6%), one palmitic, one oleic and one linoleic (P, O, L) (13.6%) [8,39]. In addition, Yousfi et al. (2009) [40] have reported one palmitic and two oleic acids (P, O, O) as one of the major acyglycerols of argan oil at a concentration of 11.42%. Stereospecific analysis determined by the use of Brockerhoff’s method shows that saturated fatty acids (palmitic or stearic) as a substitute for the external glycerol positions (Sn-1 and Sn-3), linoleic acid on the other hand occupied the position of glycerol secondary alcohol (Sn-2). Oleic acid can be found at any of these positions [39].

A more sensitive and accurate methodology is to use high resolution ^13^C-Nuclear Magnetic Resonance (^13^C-NMR) spectroscopy to characterize in depth the precise profile and regiospecificity of fatty acids in triacylglycerols [41]. In 2008, we applied this technique to argan oils [13,15], and the results indicated that the method is more convenient, less time consuming, more precise and avoids long chromatographic techniques. Thus, in ^13^C-NMR, the resonance frequency of the carbonyl is different from what can be seen in Sn1,3 compared to Sn2 and also depends on the type of fatty acids, which are evidently influenced by the electronic environment. Carbonyl spins are effectively able to discern between saturated acyl chains from their unsaturated homologs and in addition enables the determination of the position of esterification. The results are summarized in the Table 1.

#### 2.1.4. Argan Oil Phytosterols

Plant sterols or phytosterols are vegetable lipids. They are found in the membrane of plant cells, in fruits and vegetables, but especially in nuts and oil seeds. They are structurally related to cholesterol. Phytosterols are high-melting solid alcohols (steroid means solid), which can be esterified with fatty acids of vegetable origin. The argan oil sterol fraction is composed mainly of four derived sterols: Stigmastanes, consisting of δ7-sterols and are mainly spinasterol and schottenol (about 88% of the sterol fraction) [10,16] as well as stigmasta-8,22-diene-3-beta-ol in a lower proportion [16,42]. The presence of campesterol a δ5-sterol in very low concentration (0.17 to 0.31 mg/100g oil) has also been reported [43].

**Figure 1:**
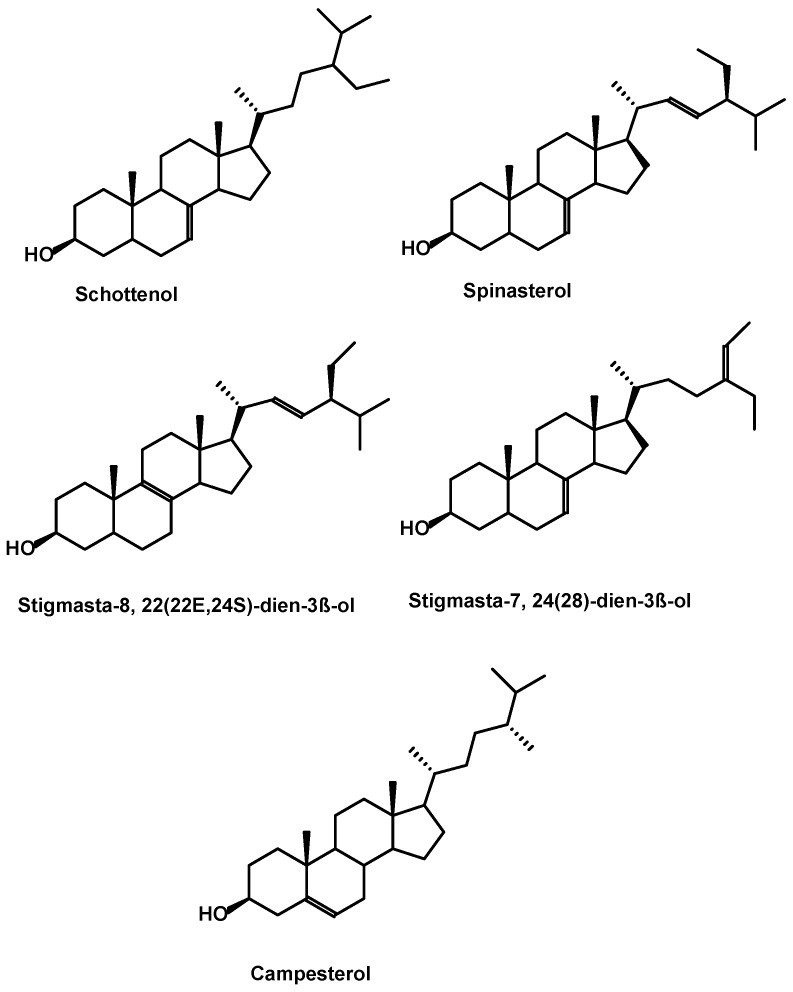
Structuresof phytosterols identified in argan oil.

Argan oil phytosterols were detected in a sterol enriched unsaponifiable fraction and were obtained from the oil after saponification with KOH (10% in methanol), i.e., under drastic conditions, which probably degrades tocopherols [16]. The main structures of the argan sterols are shown in the Figure 1.

Another point to note, argan oil (compared to other seasoning oils) contains higher levels of squalene (up to 3.2 g/kg) [16]. Hydrocarbons mainly squalene in vegetable oils are present in quantities generally lower than 0.15%, some exceptions include olive and argan oils, which exceed 0.3% as judged using two different methodologies namely on-column injection by Gas Chromatography coupled with Flame Ionization and Mass spectrometry Detectors (GC-FID and GC-MS, respectively) [16].

#### 2.1.5. Argan Oil Triterpenic Alcohols

The analysis of triterpene compounds is more delicate, due to the possible presence of numerous isomers and the absence of genuine authentic commercial standards. The unsaponifiable fractions of argan oil contain about 20% of triterpene alcohols [10].

**Figure 2:**
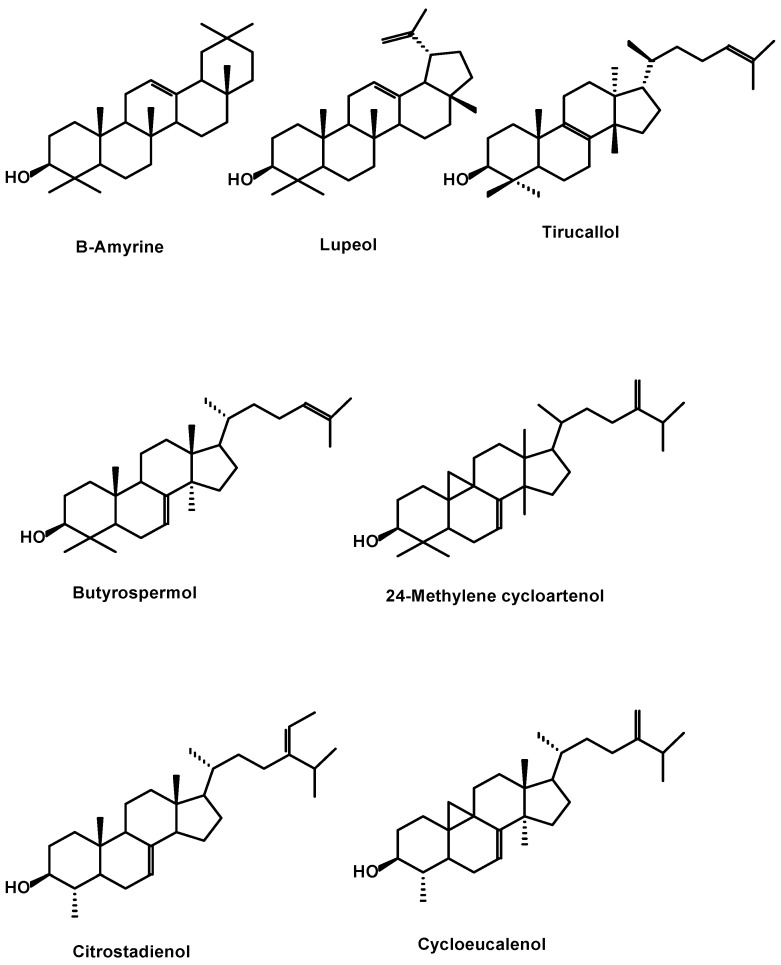
Structures of triterpenes identified in argan oil.

These are a complex group of plant constituents which consist mainly of four or five condensed cyclohexane rings containing 27–30 carbon atoms. The presence of a hydroxyl group at position C3 is inherent, and this is the key to their biogenic relationships. Triterpenes can be easily separated from sterols by chromatography. A few identified structures in crude argan oil include lupane, ursane and oleanane derivatives which include β-amyrin, butyrospermol and tirucallol as major triterpenic alcohols and represent 27.3%, 18.1% and 27.9% of the triterpenic fraction, respectively [10]. In addition, lupeol (7.1%) and 24-methylene cycloartanol (4.5%), and two methylsterols, citrostadienol (4-α-methylstigma-7,24-28-diene-3β-ol) (3.9%) and cycloeucalenol (≤5%), have also been reported [44,45]. The structures are shown in Figure 2.

#### 2.1.6. Tocopherols in Argan Oil

**Figure 3:**
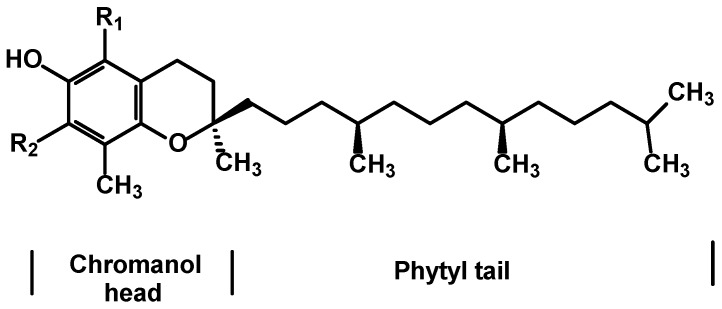
Structures of tocopherols identified in argan oil.

Tocopherols (vitamin E) are a series of fat-soluble vitamins (α, β, γ and δ) which are chiral organic molecules and vary in their degree of methylation of the chromanol nucleus. α-Tocopherol is the form of tocopherol that is the most bioavailable in humans, but the most abundant in the diet is by far γ-tocopherol (Figure 3). Vitamin E in argan oil was studied earlier but updated and quantitated unequivocally using hyphenated techniques [16]. The γ-form is actually the predominant vitamer coupled with the absence of tocotrienols in argan oils from different varieties [16]. The content of tocopherol vitamers of argan oil was determined not only by a GC-MS method but also by reverse-phase High Performance Liquid Chromatography (HPLC), the latter method is largely validated in the literature, with an isocratic eluent (MeOH) and detection at 290 nm, which allows separation of the different forms of the tocopherols in less than 8 min. Compared to olive oil and the sunflower oil the γ-form is predominant in argan oil while olive oil and sunflower oil are rich in the α-form [16].

#### 2.1.7. The Polyphenols of Argan Oil

Polyphenols are chemicals comprising at least one aromatic ring, and one or more hydroxyl groups, with molecular weights ranging from monophenols to highly polymerized compounds of more than 30,000 Daltons, and having, besides the usual properties of phenols, the ability to precipitate alkaloids, gelatin and other proteins [46].

Polyphenolics are present in very minor concentrations in argan oil, not exceeding 5 mg/kg of oil [16], represented by glycosylated ferulic acid (around 3 mg/kg alone), in association with syringic acid, vanillic acid, tyrosol, vanillin and *p*-hydroxybenzaldehyde. Other structures at the limit of detection in GC-MS, with probably non-polyphenolic structures, exist in the oil but their structures as have never been identified [16]. In addition, Rojas et al. (2005) and depending on the oils origin, detected protocatechic acid, resorcinol, (+)-catechin and (−)-epicatechin in food argan oil but not in the aesthetic form of the oil [47].

#### 2.1.8. Melatonin and Coenzyme CoQ(10) from Argan Oil

**Figure 4:**
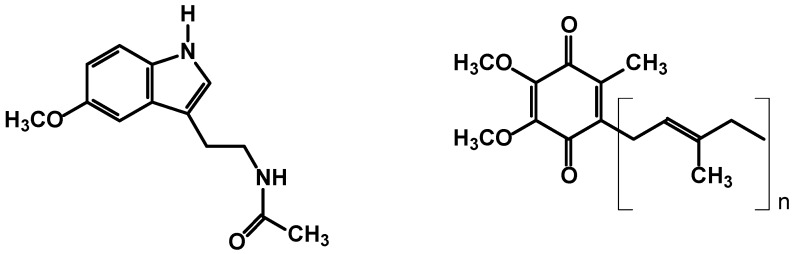
Structures of melatonin and Coenzyme Q10identified in argan oil.

Virgin argan oil possesses high antioxidant capacity, which may be partially explained by its high content of antioxidant molecules such as polyphenols and tocopherols. Virgin argan oil is in addition a rich source of CoQ(10) and melatonin [48]. The structures are depicted in Figure 4.

### 2.2. Argan Flesh Essential Oils

Among the volatile substances from the fruit pulp of the argan tree, resorcinol (73.5%) was identified as a major compound [49]. In more recent studies, the composition of the essential oil from the fresh and dried pulp of the fruit has been described and camphor (35%) was described as the major component in both oil types. In addition, the fresh fruit essential oil contains significant amounts of 1,8-cineole (16%). Others constituents are endo-borneol (12%) and 2-(4-methylcyclohex-3-enyl) propan-2-ol (11%) [50].

#### 2.2.1. Argan Flesh Triterpenes

The unsaponifiable fraction of argan fruits contains a variety of components including triterpenoids. The triterpenoids such as erythrodiol, lupeol, α- and β-amyrine, taraxasterol, ψ-taraxasterol, betulinaldehyde and betulin have been reported [8,9].

**Figure 5:**
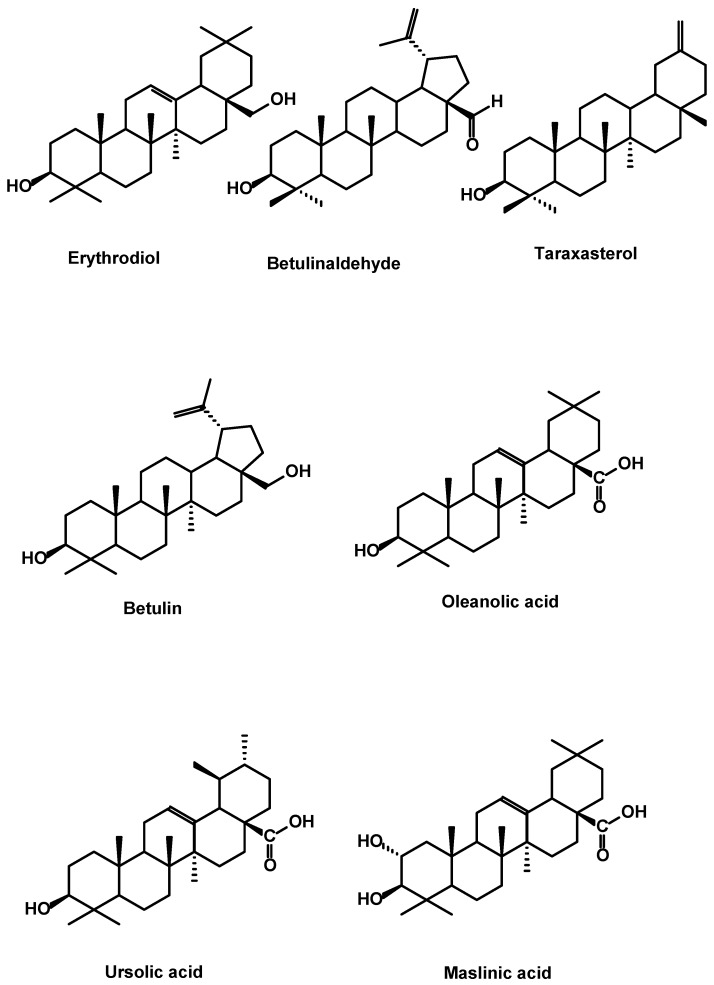
Structures of the triterpenes identified in argan flesh.

Pentacyclic triterpenic free acids in fruit pulp of *Argania spinosa* have also been reported. Ursolic acid is the major triterpene acid at about 0.44% dry weight (DW) followed by significant amounts of oleanolic acid (0.19% DW) along with minor amounts of maslinic acid [51]. The structures are shown in Figure 5.

#### 2.2.2. Argan Flesh (Pulp) Sterols

The sterols in the flesh (pulp) are extremely minor containing schottenol and spinasterol [9], whereas they are present in reasonable quantities in the oil unsaponifiable fraction.

#### 2.2.3. Argan Pulp Polyphenols

(+)-Catechin, (−)-epicatechin, rutin, and *p*-hydroxybenzoic acid were the first polyphenols to be described in the fruit pulp of *A. spinosa* [52].In addition, Charrouf et al. (2007) described further structures such as gallic acid, protocatechuic acid, rhamnetin-*O*-rutinoside, isorhoifolin, hesperidin, hyperoside, isoquercetin, naringenin-7-*O*-glucoside, quercetin-3-*O*-arabinoside, naringenin, quercetin, luteolin and aunspecified procyanidin dimer [53].

More comprehensive studies, including the precise quantitation of polyphenols in argan flesh fruits, have been conducted recently [11,12]. The method of their identification entailed Soxhlet extraction of dried pulp argan fruit, solid-phase extraction (SPE) cleanup, analysis by means of hyphenated techniques such as High Performance Liquid Chromatography coupled with Diodes array and Electrospray Mass Spectrometry Detectors (HPLC–ESI-DAD-MS) together with nano-ESI-MS–MS to confirm the identity of the polyphenolic compounds. In selected cases for more complicated structures, NMR analysis was also deemed necessary to fully validate identification.

**Figure 6:**
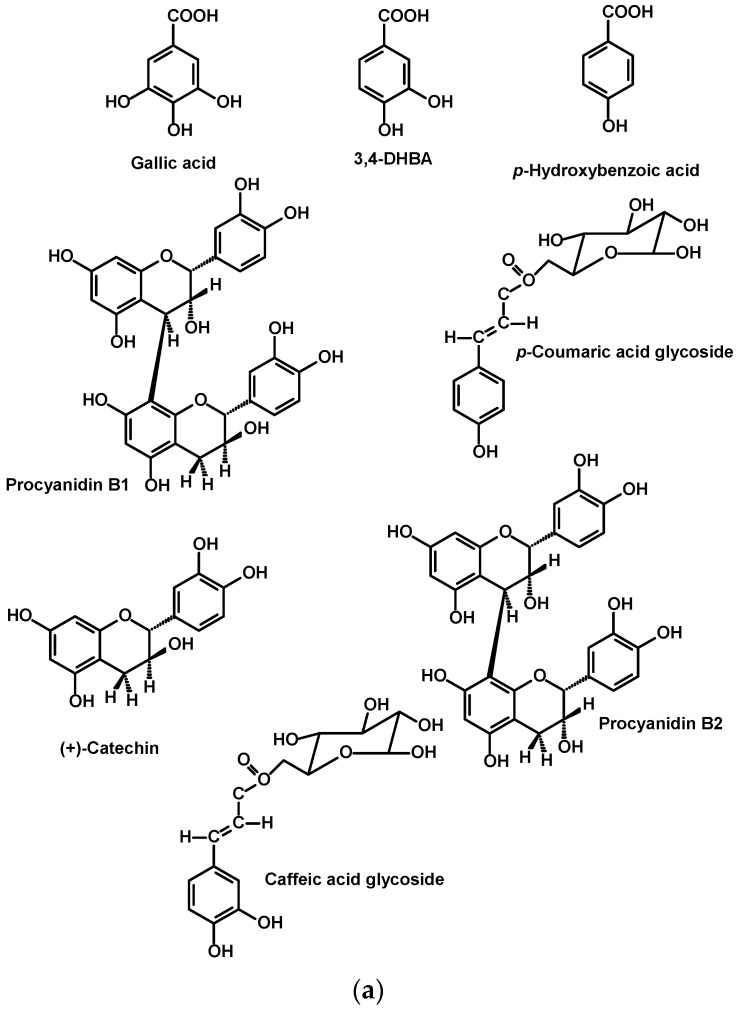

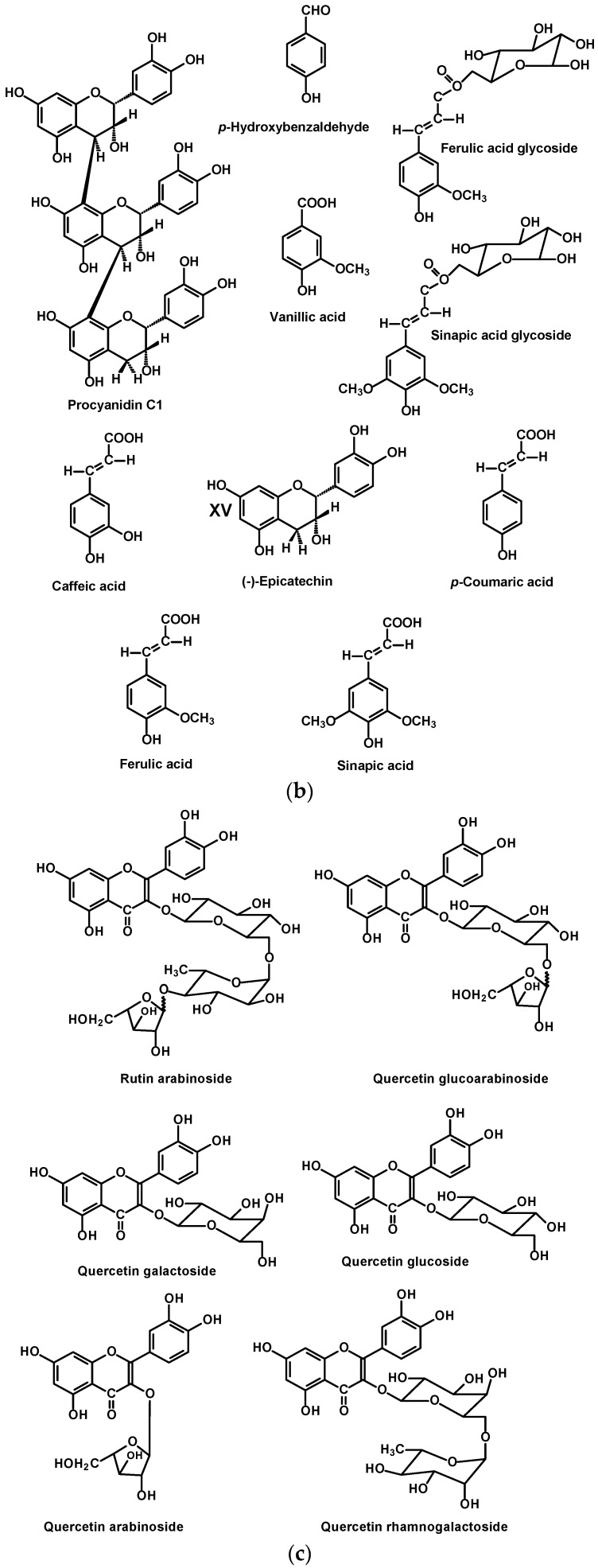

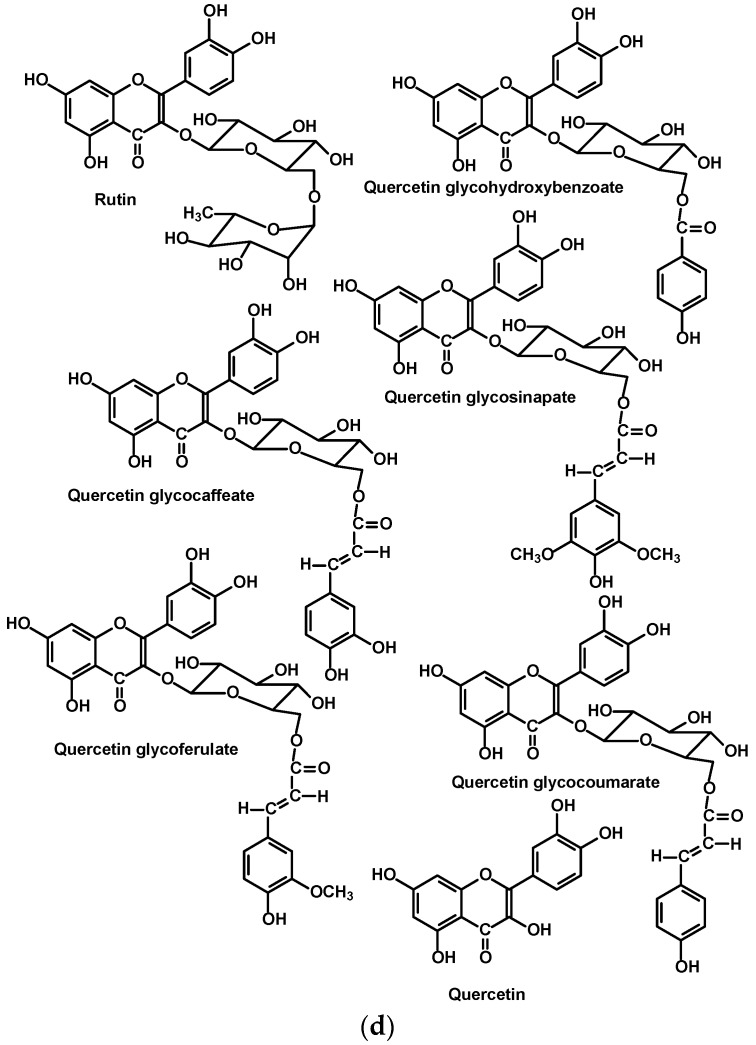
Structures of the polyphenols identified in argan flesh. (**a**,**b**) represent phenolic acids, cinnamic acids, catechins and procyanidins derivatives; (**c**,**d**) represent flavonoids and their acylglycosylated derivatives.

In these reports, thirty-two polyphenolic compounds from immature argan fruits were characterized. A total of 15.4 g/kg was determined, represented by catechins (39%), flavonoids [28%; comprised of glycosylates (20.97%), glycophenolic acids (6.60%), and free flavonoids (0.74%)], procyanidins (26%), free phenolic acids (6%), and phenolic acid glycosides (1%). The structures are shown in Figure 6a–d. These are:gallic acid, 3,4-dihydoxybenzoic acid (3,4-DHBA), epicatechin-(4*β*→8)-catechin dimer (procyanidin B1), *p*-coumaric acid glycoside, (+)-catechin, epicatechin-(4*β*→8)-epicatechin dimer (procyanidin B2), *p*-hydroxybenzoic acid, caffeic acid glycoside, epicatechin-(4*β*→8)-epicatechin-(4*β*→8)-epicatechin trimer (procyanidin C1), *p*-hydroxybenzaldehyde, ferulic acid glycoside, vanillic acid, sinapic acid glycoside, caffeic acid, (−)-epicatechin, *p*-coumaric acid, ferulic acid, sinapic acid, rutin arabinoside, quercetin glucoarabinoside, quercetin-3-*O*-galactoside, quercetin-3-*O*-glucoside, quercetin arabinoside, quercetin-3-*O*-rhamnogalactoside, quercetin-3-*O*-rhamnoglucoside (rutin), quercetin glycohydroxybenzoate, quercetin glycocaffeate, quercetin glycosinapate, quercetin glycoferulate, quercetin glycocoumarate and quercetin [11,12].

#### 2.2.4. Argan Press-Cake Polyphenols

Some known polyphenolics have also been characterized in the press-cake, and these include: catechol, resorcinol, 4-hydroxybenzyl alcohol, vanillin, tyrosol, *p*-hydroxybenzoic acid, vanillyl alcohol, 3,4-dihydroxybenzyl alcohol, 4-hydroxy-3-methoxyphenethylalcohol, methyl-3,4-benzoate, vanillic acid, hydroxytyrosol, 3,4-dihydroxybenzoic acid (protocatechuic acid), syringic acid, (−)-epicatechin, and (+)-catechin [47].

#### 2.2.5. Argan Press-Cake Saponins

Saponins were isolated and identified in the press-cake of argan fruit in 1888 and the mixture was assigned the name arganine [54]. Saponins are triterpenic or steroid heterosides, characterized mainly by their surfactant potency, which form foaming solutions on contact with water. With some exceptions, steroidal saponins are characteristic of monocotyledons, while triterpenic ones are characteristic of dicotyledons which include the Sapotaceae family.

**Figure 7:**
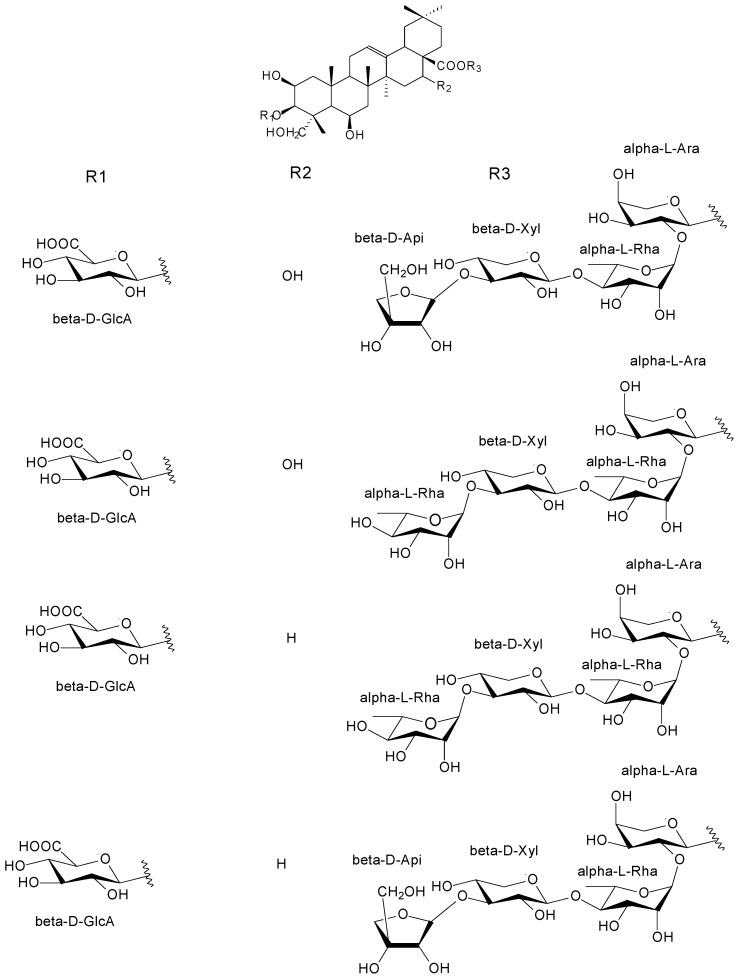
Structures of the arganines identified in argan kernel cake.

A similar saponin aglycone is found in all botanical parts of argan fruit. Saponins from hull, pulp and cake differ only slightly in the nature, number or linkage of the sugar moiety. Depending on the linking mode, argan triterpene sub-families have been determined first as “Δ12-oleane”, in particular they are derived from bassic acid (a dihydroxydienoic acid of triterpene series such as oleane). Argan saponins are characterized as protobassic as well as 16-α-hydroxyprotobassic acids where the R2 group can be either H or OH (Figure 7). These saponins are bidesmosidics (containing two sites of glycosylation). In argan kernel saponins, the linkage is linear at positions C3 and C28 and is formed mainly with glucose and a pentasaccharide made up of arabinose, xylose and three rhamnose sugars, respectively. Seven saponins have been characterized and assigned the names arganine A–F to recall their botanical origin along with already known saponin, mi-saponin [9] as well as in the shell, another saponin termed arganine K [55].

In more recent works from the argan kernel cake, saponins reported are derived from protobassic acid as well as from 16-α hydroxy-protobassic acid [56]. They have been isolated, purified and characterized, and these are also bidesmosidic at positions C3 and in the position of the ester (C28), respectively. The saponins in the kernel cake were extracted using 50% ethanol in water and due to the high polarity of the solvent, saponins with glucuronic acid moieties at the C3 positioninstead of glucose were isolated and identified. These are also oleanic-type triterpene carboxylic acid 3,28-*O*-bidesmosides assigned the names GOTCAB [56]. The structures are given in Figure 7.

Pulp, kernel and hull of argan fruits are low in saponins compared to wood (trunk) which contains also bayogenine heteroside as identified by Oulad-Ali et al. [57]. The saponin concentration in the pulp is only 0.02% [55].

#### 2.2.6. Evidence of a New Family of Secondary Metabolism in the Argan Flesh Fruits

Our research on argan flesh polyphenols resulted in the discovery of the following new secondary metabolites namely arganimides and argaminolics, of which arganimide A is described in Argania for the first time and argaminolics in the plant kingdom for the first time [11,12,17,18].

They include, to date, arganimide A (4,4′-dihydroxy-3,3′-imino-di-benzoic acid), a special type of phenolic compound containing a nitrogen atom as a diphenyl amine derivative which belongs to an obscure and extremely rare class of natural products. From data already reported in the literature, arganimide A (4,4′-dihydroxy-3,3′-imino-di-benzoic acid) has been nevertheless reported as a natural product only once before [58]. A related structure, ostensibly *N*-(3,4-dihydroxybenzoyl)-3,4-dihydroxybenzamide isolated from *Pu-erh* tea but with virtually identical NMR spins to arganimide A identified in argan fruits has also been described [59].

Similar aminophenols were first described in oats (*Avena sativa*) [60], and the bacterial species streptomyces [61,62]. These were assigned avenanthramides (AVAs), but their structures are quite different from those detected in the fruits of argan. These metabolites are a group of alkaloid phenols consisting of anthranilic acid, and hydroxycinnamic acid derivatives linked by a pseudo-peptide bond, where the substitution pattern on both parts is what distinguishes the different avenanthramides from one another. The three hydroxycinnamic acids mostly involved are *p*-coumaric acid for AVA-A, ferulic acid for AVA-B and caffeic acid for AVA-C.

**Figure 8:**
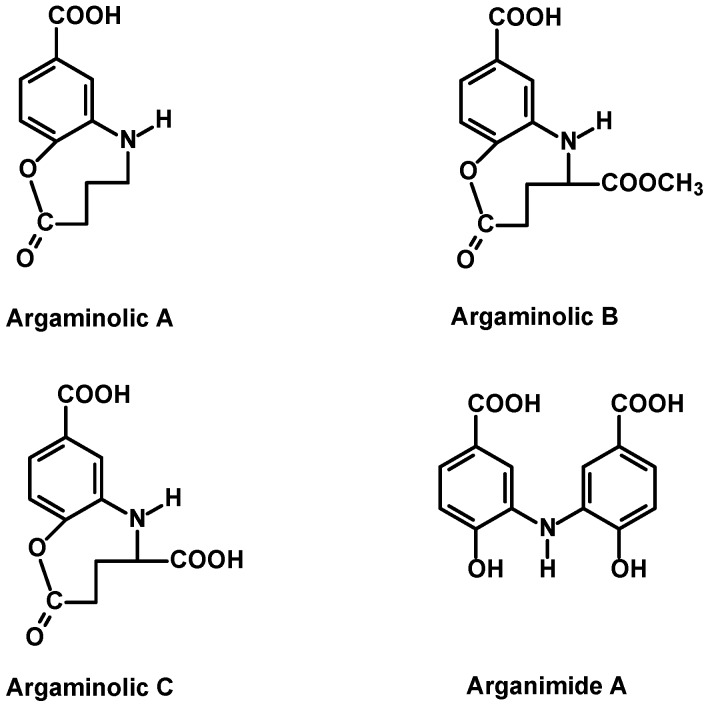
Aminophenols identified in argan fruit flesh.

The second new series of argan fruits namely the argaminolics have structures closely linked to nuclei based on benzoxazocines derivatives [12,17,18]. The natural compounds reported here consist of a carboxylic analog called argaminolic B, and a dicarboxylic analog named argaminolic C as well as achiral argaminolic A (Figure 8). These are also very unusual structures containing fused bicyclic ring structures, of which an analog substance has been characterized in *Peristrophe lanceolaria* only [63].

#### 2.2.7. Carbohydrates

Polysaccharides, including hemicellulose, are well exploited by the food, cosmetic, and pharmaceutical industries for their valuable properties. Hemicelluloses constitute the most abundant polysaccharide after cellulose in nature. These are alkaline-soluble polysaccharides comprising pentoses, hexoses and glucuronic acid derivatives [64], the major neutral hemicellulosic polysaccharide in argan pulp is a galacto-xyloglucan and also shows that fruit pulps is a rich source of arabinogalactan-proteins [65].

#### 2.2.8. Polyisoprenes

A drop of latex appears when the cuticle that covers the pulp itself is pierced. Argan hulls contain latex and this rubber fraction consists of *trans*- and *cis*-poly-isoprenic structures, which were confirmed by Fourier Transform Infra Red and ^13^C- and ^1^H-Nuclear Magnetic Resonance spectroscopy [66].

#### 2.2.9. Argan Fruit Health Benefits

Argan fruit is considered to have several health beneficial effects. It furnishes, after extraction, edible, dietary and industrializable oil with a hazelnut taste and brown color. Argan oil, with its well-balanced composition of fatty acids, provides in addition an appreciable amount of biologically active minor components. Literature data certifythat lipid consumption is positively correlated with the induction of several chronic diseases, however, epidemiological data have shown that occurrence of these pathologies depends not only on the amount of lipids consumed, but also on the type of fatty substances which are important in their etiology [67,68,69,70].

Argan oil does not contain essential omega-3, but it is a rich source of monounsaturated oleic and omega-6 linoleic acids (more than 80% of the total oil weight). From a nutritional point of view, these fatty acids are equivalent to peanut or sesame oils in terms of their lipid composition. A growing body of literature has been published on the health benefits of oleic acid [71,72,73,74,75].

In addition, argan oil contains barely detectable levels of trans vaccenic acid (TVA; trans-11 18:1), a positional and geometric isomer of oleic acid [13] which is usually encountered in animal fats. This isomer has many beneficial effects including inhibition of the proliferation of MCF-7 cells by down-regulating the expression of Bcl-2 as well as procaspase-9 [76].

In other respects, triacylglycerols are the first fat that impact lipid metabolism in the human body, and consequently the structures and the positions of the fatty acids in the triacylglycerols are very important to elucidate their function. Gut pancreatic lipases hydrolyze fatty acids in external positions, thus Sn1,3 acylglycerols are absorbed after hydrolysis into free fatty acids (FFA) and metabolized, unlike Sn2 monoglycerides (MG) which are absorbed intact.

Furthermore, argan oil contains a substantial concentration of γ-tocopherol detected at levels up to 75% of total tocopherols [16]. Previous in vivo data indicate that γ-tocopherol may be a much more potent antioxidant than α-tocopherol. It is highly likely that γ-tocopherol unlike α-tocopherol will protect against reactive oxygen species mediated inflammation [77].

In vivo, tocopherols are incorporated into cell membranes and their antioxidant capacities (unlike phenolic antioxidants, of which a continuous supply is needed) are modulated by the concentration of vitamin C, which make their pharmacology rather different from polyphenols and their half-life extended. However, other works conducted thus far do not provide any convincing evidence to recommend supplementation of α-tocopherol as a public health measure since contradictions are feared [78].

The sterol content of argan oils is in the range 272–357 mg/100g [16]. These sterols are rare in vegetable oils, and interestingly, their occurrence in argan oil and the absence of β-sitosterol, which is present in several other vegetable oils is a bona fide marker of argan oil adulteration with other cheaper ones mostly containing β-sitosterol.

From a phytopharmacological point of view, in preclinical studies, spinasterol has been shown to reduce plasma and liver cholesterol levels of mice, modulated by increasing faecal cholesterol excretion [79]. An antitumorigenic effect has been demonstrated by Villasenor and Domingo (2000) [80], whereas schottenol also exhibits anticarcinogenic and cytotoxic potential [81]. More recently, the effects of these phytosterols on cell growth and mitochondrial activity were evaluated on three cell lines, 158N murine oligodendrocytes, C6 rat glioma cells, and SK-N-BE human neuronal cells. The effects of spinasterol and schottenol were compared with 7-ketocholesterol and ferulic acid, which is also present in argan oil. The results suggest that spinasterol and schottenol can modulate mitochondrial activity and influence cell metabolism [82].

Schottenol and spinasterol are also considered as new Liver X Receptors (LXR) agonists, which may play protective roles by the modulation of cholesterol metabolism [83].

Argan oil can be used as an emollient and as a source of beneficial ingredients for cosmetics such as squalene (acyclic triterpene that is an intermediate in cholesterol biosynthesis) with relatively high content (0.3%) [16], and is believed to be a protective cosmeceutical agent against skin cancer and is reported to be the primary antioxidant for scavenging superoxide radicals formed by photosensitized oxidation due to UV rays on the surface of the skin [84,85].

Other researchers have shown that squalene is an effective inhibitor of chemically-induced colon, lung as well as skin tumourigenesis in rodents. This protective effect is observed when squalene is given before and/or during carcinogen treatment and the mechanisms involved for its chemopreventive activity may include inhibition of Ras farnesylation, modulation of carcinogen activation and antioxidant activities [86].

In other reports, squalene enhances excretion of xenobiotics [87], is a cholesterol-lowering drug, inhibits cholesterol biosynthesis through inhibition of HMG-COA reductase, squalene synthase and limits in addition cholesterol absorption [88] as well as increasing high density lipoprotein-cholesterol and paraoxonase 1 [89].

Argan oil is also a rich source of triterpenoids including lupane, ursane and oleane subtypes, and their health beneficial effects have been well documented. There is evidence of their anti-cancer, anti-allergic, anti-inflammatory, antiviral (including anti-HIV), anti-bacterial, anti-fungal, hepatoprotective, anti-ulcer, hypolipidemic, anti-atherosclerotic, and anti-hyperglycemic-diabetic effects [90,91,92].

Overall, most of the traditionally claimed activities of argan oil have been confirmed scientifically; thus, argan oil has been reported to inhibit platelet aggregation coupled with enhancement of oxidative status [93]. Another study emphasizes the importance of its antithrombotic potential at a dose of 1 mL/100 g/day [94].

Research provides also evidence that argan oil has an undeniable anti-carcinogenic effect by preventing human prostate cancer. Tocopherols and saponins extracted from the argan tree exhibited a dose-response cytotoxic effect and an anti-proliferative action on DU145 and LNCaP cell lines (28 and 32 μg/mL, as IC_50_) respectively [95].For the PC3 cell line, the best anti-proliferative effect was obtained by argan sterols with an IC_50_ = 43 μg/mL [96].

Some other recent preclinical reports in rat models have shown that argan oil prevented the body mass loss, induced a significant reduction of blood glucose and a significant increase of hepatic glycogen level as well as modulate insulin-resistance [97,98]. Argan oil intake reduced blood pressure, hyperglycemia and insulin resistance through its antioxidant properties as reflected by the normalization of the superoxide anion production and the NADPH oxidase activity at the vascular level in one nutritional hypertensive insulin-resistant rat model [99]. A 50% decrease in the risk of developing arterial hypertension, a 37% decrease in blood cholesterol, 67% in LDL, 31% in triglycerides and 13% in body weighthas also been reported [100,101].

In therapeutic applications, argan oil rich in omega-6 polyunsaturated fatty acids have been studied as a mean for delivery of lipophilic cytotoxic antineoplastic agents. With its use, several promising nanoemulsion formulations with extended shelf-life and stability have been designed. Argan oil nanoemulsions, stabilized with various emulsifying mixtures of polyethoxylated solutol HS-15 and polyethyleneglucol vitamin E succinyl ester (TPGS), demonstrated significant pro-apoptotic effect on murine breast and colon carcinoma cell lines [102].

Some few clinical studies provide evidence that argan oil can promote additional benefits on risk factors for cardiovascular disease. In 2005, sixty young men were included in an interventional study. The analysis of the data indicated an increase of paraoxanase 1 (PON1) activities coupled with an increase of plasma vitamin E concentration [103]. Some years later, the vitamin E serum level of 151 menopausal women consuming either olive or argan oil was determined, and in the argan oil consumer group, Vitamin E serum level was increased [104]. In another study carried out on 60 young and healthy male volunteers aged between 23 and 40 years old, consumption of argan oil showed, after three weeks of intake, a significant increase in serum concentration levels of the androgen hormones includingtestosterone and luteinizing hormone [105]. More recently, 37 patients (18 men, 19 women) with end-stage renal disease on maintenance displayed an improvement in markers directly associated with the lipid profile and oxidative stress status [106]. In addition, evidence from sixty menopausal women indicates that the daily consumption and/or topical application of argan oil for a period of 1–2 months has an anti-aging effect on the skin demonstrated by the improvement of skin elasticity [107].

Otherwise, various epidemiologic studies have linked polyphenol consumption with chemoprevention of certain cancers, strokes and cardiovascular diseases among other chronic maladies. Some polyphenol bioactivities, include inhibition of the DNA oxidation marker (8-hydroxydesoxy-guanosine), metal chelation which delays proxidant effects [108,109], modulation of cellular signaling pathways, inducing glutathione biosynthesis among other well known cell processes. In particular polyphenolics offer cell protection through expression of nuclear factor erythroid 2-related factor 2 (Nrf2, a basic helix–loop–helix transcription factor) as well as activating signal-transduction pathways through NF-κB and activated protein-1 AP-1 DNA binding [110,111,112].

Studies using HT-1080 brosarcoma and MSV-MDCK-invasive cells have confirmed the anti-proliferative activity of argan oil polyphenols [113].

Unsaponifiable fractions of argan oil are a rich source of triterpenes, which are ubiquitous in the plant kingdom and afford a number of beneficial effects against human diseases, including anti-inflammatory, anti-proliferative, and pro-apoptotic, anti-angiogenesis, and chemopreventive autophagy. This in fact demonstrate significant power to control manifold cancer signaling cells and physiologic processes, including NF-κB, Wnt/β-catenin, PI3K/Akt, apoptosis, among many other pathways related to cell death or cell uncontrolled proliferation. Examples of triterpene anticancer potential against colon, breast, prostate, and melanoma are reported in [114,115].

Melatonin and Coenzyme Q10have been also characterized in argan oil [48], and it was reported that their antioxidant effects have consideration on apoptosis and oxidative damage caused by single-dose ochratoxin A in rat kidney [116].

As regards to argan pulp, this is described to contain essential oils and an unsaponifiable fraction (up to 3.3%) which is rich in sterols and tritepene aglycones. Some compounds have already found industrial applications such as erythrodiol [117]. Glycosylated triterpenes (saponins) of the basic acid-type in the argan pulp have been assayed in a few preliminary biological assays and deserve special attention related to their pharmacology. These are more particularly fungicidal against *Cladosporium cucumerinum* and *Polysticus versicolor*, antibacterial as well as analgesic and anti-inflammatory agents with low toxicity. Molluscicidal activity against *Biophalaria glabrata*, has also been reported [118].

Argan pulp, contains a diverse range of polyphenols with flavans, procyanidins, flavonoids (mostly quercetin derivatives), phenolic acids along with a minor phenolic aldehyde being reported at a concentration of up to 15.4 g/kg [12].

The acyl-glucosylflavonoids which are also present in argan pulp have shown a variety of functions, such as anti-feedant and phytoalexin capacity, signaling molecules, and UV protectants [112]. Acyl-glucosylpolyphenols are physiologically important phenolic compounds in plants because these are the final, functional products of secondary metabolism [119].

Ripe argan fruit flesh is a very rich source of an unusual class of aminophenols at about 80% of the total extract [11]. 4,4′-dihydroxy-3,3′-imino-di-benzoic acid(assigned the name arganimide A) is the major compound in this family [11]. A major difference between mature and immature fruits is that phenolic acids, flavans and procyanidins virtually disappear in mature fruits. The proportional representation of argan pulp polyphenols reported for immature fruits was, catechins and procyanidins (56%), flavonoids (24%), amino phenols (13%) and phenolic acids (6%) Consequently, the proportional representation of polyphenolic compounds, in the mature fruits changed [11] dramatically to amino phenols (79%), phenolic acids (10%) and flavonoids (11%). Future studies are recommended to unravel this phenomenon. Altogether, this family of aminophenols appears to be unique to argan fruits and thus represent a bona fide chemotaxonomic marker for this species.

Earlier in the literature, some aminophenols with structures similar to arganimide A, termed avenathramides were detected and characterized only in oatmeal (*Avena sativa*). The Food and Drug Administration (FDA) recognized colloidal oatmeal as safe and effective, and granted approval as a skin protectant [120]. The avenanthramides were reported (unlike other secondary metabolites) to be the most potent at reducing UV-induced erythema 24 h after dermal application at very low concentrations ranging from 45 ppm to below 2 ppm.

Avenanthramides are 10 to 30 times more potent antioxidants than other non-amidated oat phenolic homologs and almost 20% more antioxidant than *α*-tocopherol in a linoleic acid-oxygen consumption system [121]; theyinhibitprostaglandin biosynthesis and inhibit the release the pro-inflammatory cytokines that are subsequently useful for relieving itch, sunburn, and skin inflammation. In vitro, this activity was equivalent to the positive control indomethacin [122,123].

Avenanthramides are bioavailable and accumulate in hepatic, cardiac, and skeletal muscle tissue, following oral gavage in rats [124] and may offer indirect protection by activating endogenous antioxidant defense systems including superoxide dismutase (SOD) activity of red blood cells [125].

In human kidney cells, oat avenanthramides induce heme oxygenase-1 expression via Nrf2-mediated signaling in HK-2 cells [126] and prevent osteoblast and osteocyte apoptosis and induce osteoclast apoptosis in vitro in aNrf2-independent manner [127].

In preclinical trials, diets high in oat beta-glucan activated the gut-hypothalamic (PYY(3)(−)(3)(6)-NPY) axis and increased satiety in diet-induced obesity in mice [128]. Dietary oats modulate atherogenic pathways by elevating endothelium nitric oxide production [129]. Earlier studies found that avenanthramides decreased exercise-related free radical production in soleus muscle and malondialdehyde production in heart and also increased superoxide dismutase activity in skeletal muscle, liver, and kidneys in rats [130].

In some clinical studies, oat consumption may confer a hypocaloric intervention through caloric dilution [131], preventing obesity and reducing body fat [132,133].

The aminophenols described in argan are not only diphenylamine derivatives assigned the names arganimide A and other derivatives (unpublished data) but also a second class of metabolite belonging to benzoxazocine derivatives and assigned the names argaminolics. A similar compound was first isolated from the plant *Peristrophe lanceolaria* (Acanthaceae) which is widely distributed in the temperate and tropical regions of Africa and Asia, especially in northern Thailand where it has been used as a traditional medication to treat glossitis, smallpox, infection injuries, rashes and fever [63].

Besides these new natural products, a synthetic analog structure (Nefopam), which is derived from benzoxazocine was developed in the 1960s and is a centrally acting, non-narcotic and non-steroidal analgesic drug. Intraoperative Nefopam infusion during laparoscopic cholecystectomy reduced opioid requirements and pain during the early postoperative period [134]. However, the mechanism of analgesic action is not understood, but is similar to that of monoamine reuptake inhibitors (serotonin, norepinephrine, dopamine and noradrenaline) and is used not only as an analgesic drug for nociceptive pain, but also as a treatment for the prevention of postoperative thrill and hiccups. In some reports, Nefopam blocks voltage-sensitive sodium channels and modulates glutamatergic transmission [135] as well as being effective in the prevention and treatment of shivering [136,137].

## 3. Conclusions

Although not essential for growth and development of the body’s major functions, nutraceuticals and cosmeceuticals may play a critical role in maintaining human health. The argan tree is a source of a number of bioactive metabolites. In this review, we emphasize the importance of the argan tree and particularly its fruit from a socio-economic and environmental point of view. Phytochemistry, ethnopharmacology and ethnobotany aspects are updated. A number of argan bioactive compounds may play an important role against several ailments including arthritis, hypertension, diabetes, skin diseases, cardiovascular disorders, and cancer.

Argan flesh (pulp) contains a broad spectrum of polyphenolic compounds which may have utility for incorporation into nutraceuticals and cosmeceuticals relevant to the food, cosmetic and health industries. Further research is recommended, especially on the health beneficial effects of the aminophenols.

In addition, a number of studies have determined the beneficial effects of argan oil and its byproducts against cardiovascular disease, cancer and diabetes. However, the molecular basis for these effects remains to be elucidated. Only in vitro studies have been designed to identify how argan oil exerts its effects at the cellular and preclinical level. Research related to epidemiology (case control, and cohort studies) has to be reinforced, and care is needed to extrapolate in vitro data of argan fruits metabolites to the in vivo situations.

Other physiological phenomena, which may be influenced by the consumption of argan oil, warrant further research.

## Figures and Tables

**Table:** 

Tocol Forms	R1	R2
α	CH_3_	CH_3_
β	CH_3_	H
γ	H	CH_3_
δ	H	H

**Table 1:** Positionaldistribution of fatty acids (%) in argan oil [13,15].

Argan Oil	Food Variety (%)	Aesthetic Variety (%)
**Sn-1,3**		
Saturated fatty acids	20.5	20.7
Oleic acid	28.9	30.3
Vaccenic acid	1.0	0.9
Linoleic acid	14.4	19.4
**Sn-2**		
Oleic acid	17.2	15.3
Linoleic acid	13.9	13.3
